# Evaluation and Management of Neurogenic Bladder: What Is New in China?

**DOI:** 10.3390/ijms160818580

**Published:** 2015-08-10

**Authors:** Limin Liao

**Affiliations:** 1 Department of Urology, China Rehabilitation Research Center, Beijing 100068, China; E-Mail: liaolimin64@gmail.com; Tel.: +86-10-8756-9043; Fax: +86-10-6757-0492; 2 Department of Urology, Capital Medical University, Beijing 100069, China; 3 Center of Neural Injury and Repair, Beijing Institute for Brain Disorders, Beijing 100068, China; 4 Beijing Key Laboratory of Neural Injury and Rehabilitation, Beijing 100068, China

**Keywords:** neurogenic bladder, neurogenic lower urinary tract dysfunction, central nervous system abnormalities, peripheral nerve damage, diagnosis, treatment

## Abstract

Neurogenic bladder (NB) or neurogenic lower urinary tract dysfunction (NLUTD), a dysfunction of the urinary bladder and urethra due to disease of the central nervous system or peripheral nerves, is a major global medical and social problem. Numerous nervous system abnormalities, such as: stroke, Alzheimer’s and Parkinson’s diseases, traumatic spinal cord injury, spinal cord tumors, congenital spina bifida, and diabetes, can cause NB/NLUTD. There are two major types of bladder control problems associated with NB/NLUTD: the bladder becomes either overactive or underactive depending on the nature, level, and extent of nerve damage. This review specifically focuses on the diagnosis and management of NB/NLUTD in China as well as on recent efforts to treat this disease.

## 1. Introduction

Neurogenic bladder (NB) or neurogenic lower urinary tract dysfunction (NLUTD), a dysfunction of the urinary bladder and urethra due to diseases of the central nervous system or peripheral nerves, is a major medical and social problem. NB or NLUTD may be caused by various diseases and events affecting the nervous system controlling the lower urinary tract (LUT), including the bladder and urethra. The result of NLUTD depends on the location and extent of the neurologic lesion. The loss of supra-spinal control leads to neurogenic detrusor over-activity (NDO), thus causing urinary incontinence, and detrusor sphincter dyssynergia (DSD), which results in elevated bladder pressure during the storage and voiding phases. NDO, DSD, and high pressure often lead to structural bladder damage, vesicoureteral reflux (VUR), upper urinary tract dilation (UUTD), and renal insufficiency. Therefore, the management and treatment for NB should protect upper urinary tract (UUT) function, achieve urinary continence, improve the quality of life, and restore LUT function [1]. The current methods used to treat NB include early neuroprotection actions and special measures focusing on the LUT, including medications, botulinum toxin A (BTX-A) injection, neuromodulation, and surgical procedures. Each of the current methods used to treat NB has advantages and disadvantages. In recent years, scientific technology has progressed at a rapid rate. Indeed, we have worked to translate the progress being made in basic science into clinical medicine and improvements in established therapeutic areas, as well as investigating new treatment possibilities for NB, including tissue engineering, stem cell transplantation, and gene therapy. Herein, I review some new work and findings regarding NB and NLUTD in China.

## 2. Evaluation

### 2.1. Brain Magnetic Resonance Imaging (MRI)

**Figure 1:**
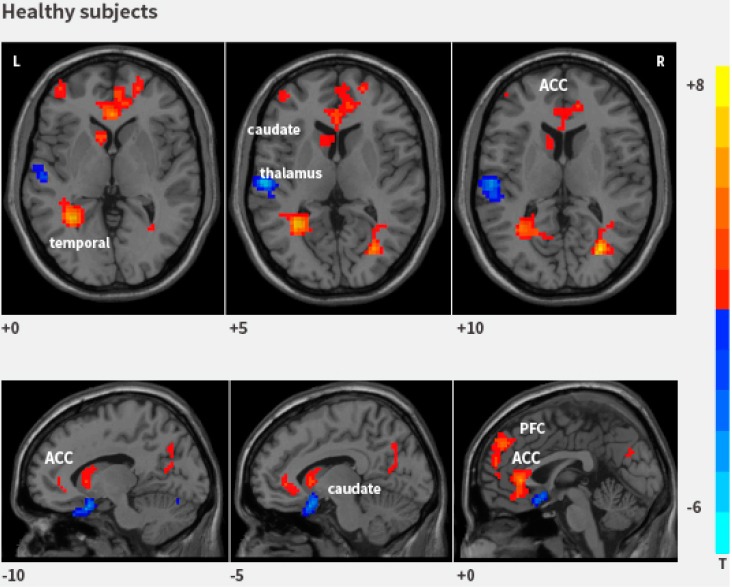
rs-fMRI maps showing statistically significant differences between the strong desire to void and the empty bladder. Regions with increased regional homogeneity (Reho) values are shown in red, and decreased Reho values are shown in blue.

Identifying brain areas involved in changes in bladder volume and urgency to void affecting brain activity might help to understand brain mechanisms that control urinary continence and micturition. It has been postulated that specific brain areas are involved in the pathophysiology underlying symptoms of storage dysfunction, such as urgency, frequency, and urge incontinence [2]. Several positron emission tomography (PET) and functional MRI (fMRI) studies have identified brain structures that are activated during bladder filling and voiding in healthy subjects and symptomatic patients [2,3,4]. These studies made use of procedures, including in-dwelling catheters or assignments during the scanning process, which potentially influenced brain activity significantly. Resting-state functional MRI (rs-fMRI) is a non-invasive neuroimaging technique used to study changes in regional cerebral blood flow (rCBF) in humans. Our results suggest that extensive brain regions are active in healthy subjects with a strong desire to void, including the frontal lobe, cingulate cortex, caudate nucleus, hypothalamus, and temporal lobes (Figure 1) [5]. Specifically, we reported two interesting results: first, activation in the temporal lobes, which are part of the default mode network (DMN), need to be studied further in pathologic conditions; and second, the different activity regions in men and women have not been previously reported. It was clearly shown that rs-fMRI can be used to study the mutual association between the bladder and the brain [5].

In our next research, gender differences in the central control of urinary bladder storage, the networks, and function connectivity in brain control using rs-fMRI will be explored. Lower urinary tract symptoms (LUTS) may result from various diseases and events affecting the central nervous system (CNS), including cerebrovascular accidents, brain tumors, dementia, and Parkinson’s disease. The location and extent of the neurologic lesions in the brain and the functional changes resulting in the brain from these lesions in NB patients will also be confirmed by rs-fMRI investigation in corollary studies.

### 2.2. History and Physical Examination

A thorough medical history and physical examination is mandatory before any additional diagnostic investigations are planned. The clinical assessment of patients with NLUTD includes a detailed history, a patient voiding diary, and a thorough physical examination. The initial evaluation is essential to determine the therapeutic scheme for long-term treatment and follow-up.

### 2.3. Urodynamics

**Figure 2:**
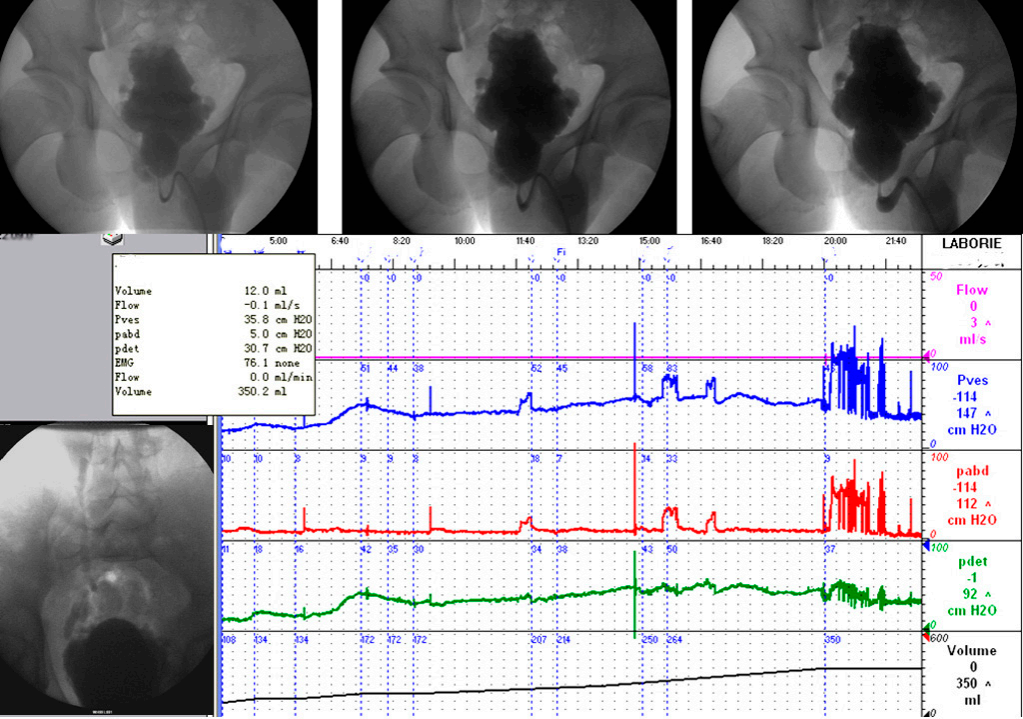
Video-urodynamics findings of patients with NB. Typical detrusor overactivity (DO), detrusor external sphincter dyssynergia (DESD), and right vesicoureteral reflux (VUR) are shown.

An urodynamic investigation is the only method that can objectively assess the function and dysfunction of the LUT. It is essential to describe the LUT status in patients with NLUTD [6]. In these patients, particularly when detrusor overactivity (DO) might be present, an invasive urodynamic investigation is even more critical than in other patients. Any technical source of artifacts must be critically considered. The quality of the urodynamic recording and its interpretation must be ensured. The urodynamic tests are performed and all urodynamic findings must be reported in detail according to the international continence society (ICS) technical recommendations and standards [6]. In previous studies, we established the typical value ranges (TVRs), described the typical signal patterns (TSPs), and outlined the roles of TVRs and TSPs in urodynamic quality control [7,8]. We also found that retrospective quality control on pressure-flow data with computer-based urodynamic systems is necessary [9]. Video-urodynamics (VUD) is the combination of filling cystometry and a pressure-flow study with imaging; VUD is the gold standard for urodynamic investigation in patients with NLUTD [10]. Possible pathologic findings demonstrated on VUD include all as described under cystometry and pressure-flow study plus the morphologic pathology of the LUT and upper urinary tract (UUT), including the DSD and VUR (Figure 2). We performed approximately 10,000 VUD studies at our center in the past 20 years.

### 2.4. Upper Urinary Tract Imaging

#### 2.4.1. Upper Urinary Tract Dilation (UUTD)

**Figure 3:**
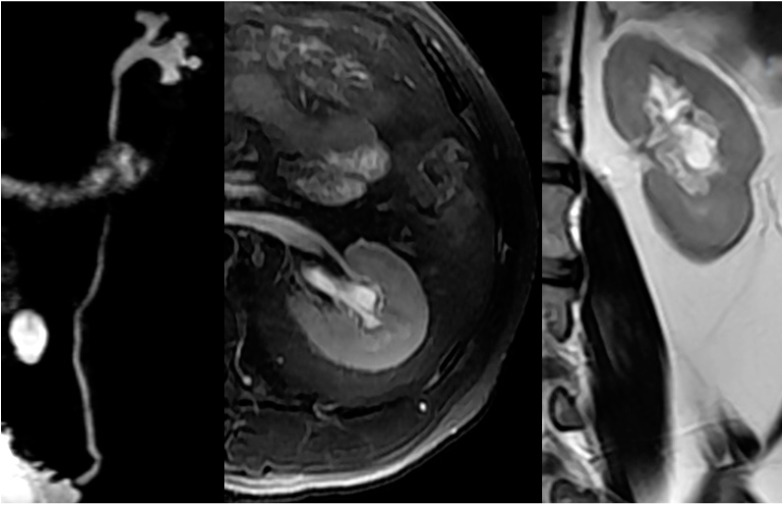
Grade 1 with UUTD-MRU grading system.

**Figure 4:**
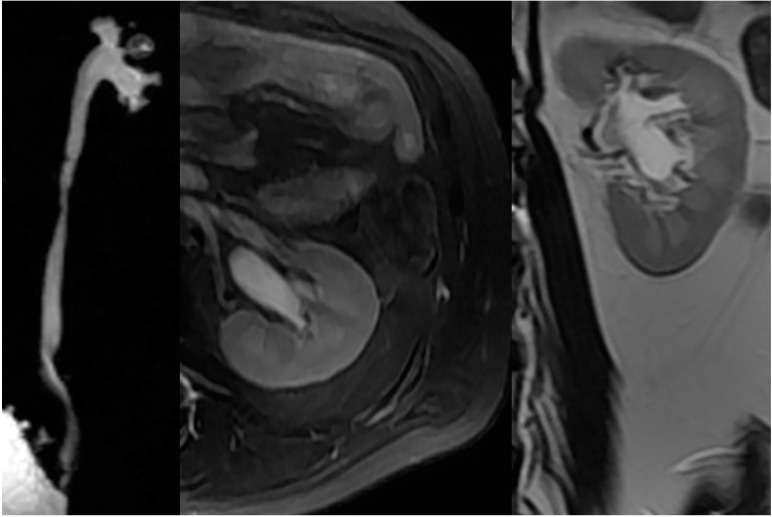
Grade 2 with UUTD-MRU grading system.

**Figure 5:**
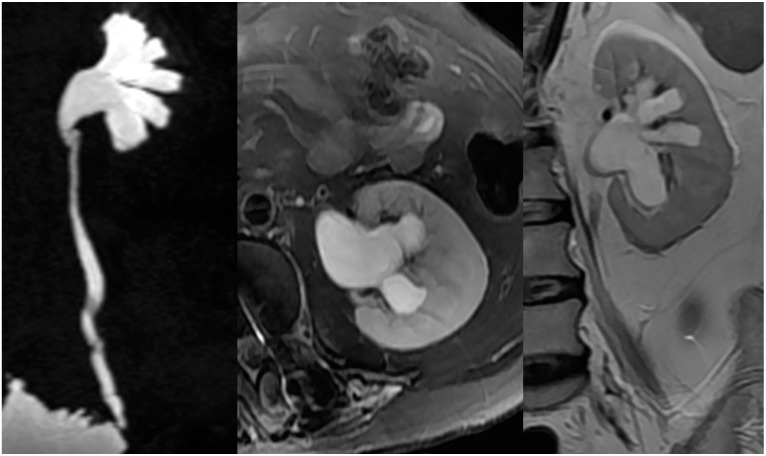
Grade 3 with UUTD-MRU grading system.

**Figure 6:**
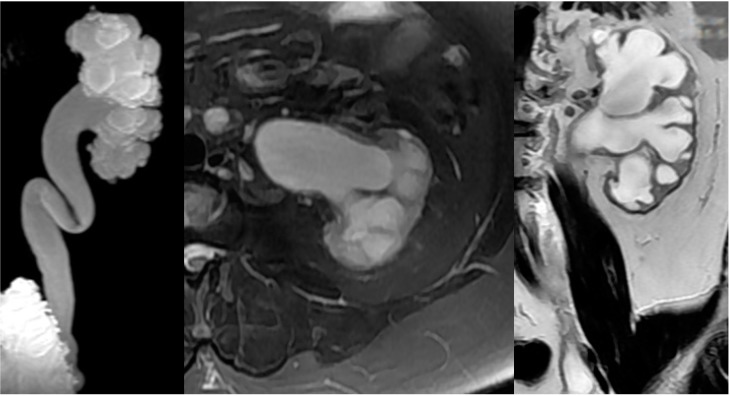
Grade 4 with UUTD-MRU grading system.

In patients with NLUTD, elevated intravesical pressures can be transmitted to the UUT causing hydronephrosis (HN) and ureteral dilation (UD), which are referred to as UUTD. Ureteral obstruction at the bladder wall is another cause for UUTD, but is less of a concern. UUTD or deterioration can lead to chronic renal failure. Therefore, evaluation and protection of UUT function is extremely important in the management of NB. Currently, the most common method used to detect HN and UD is ultrasonography (US). HN has been subjectively classified as mild, moderate, and severe. Moreover, US findings are not easy to interpret by a clinical urologist (particularly prominent issue in China) because the kidneys and ureters are not shown in the same image, and is thus a somewhat subjective assessment for UUTD. We have described a new grading system for UUTD, including HN and UD, based on magnetic resonance urography (MRU), and provided clinicians with a more objective, intuitive, and understandable new method for UUTD grading [11]. The UUTD was graded by MRU as follows: grade 0, the central renal complex is closely apposed without UD; grade 1, slight separation of the central renal complex exists, and the ureter is <7 mm in diameter (Figure 3); grade 2, the renal pelvis is further dilated, and a single or a few calices may be visualized and the ureter is <10 mm in diameter (Figure 4); grade 3, the renal pelvis is dilated, there are fluid-filled calices throughout the kidney, the renal parenchyma overlying the calices has thinned (renal parenchyma loss <50%), and the ureter is tortuous and <15 mm in diameter (Figure 5); and grade 4, similar to grade 3, but the renal parenchyma over the calices is thinned (renal parenchyma loss >50%), the ureter is severely tortuous, and the ureter is >15 mm in diameter (Figure 6). Although MRU grading is much more expensive than US grading, MRU grading has some benefits. Indeed, we have adopted MRU in our clinical practice for approximately 10 years.

#### 2.4.2. Vesicoureteral Reflux

The VUR was identified by VUD with X-ray digital fluoroscopy and graded from I–V based on the international reflux grading system (IRGS) [12].

### 2.5. Classification

The NLUTD classification provides a standardized terminology. Several classification systems have been proposed. A simple classification focusing on therapeutic consequences has been proposed by Madersbacher [13]. This classification describes several NLUTD symptoms on the basis of the contraction state of the bladder and external urethral sphincter during voiding and the filling phase. The existing classification schemes have only focused on the bladder and urethra, and are unrelated to UUTD, including HN, VUR, and UD [1,14]. In a previous study, I described a new UUTD grading system, including HN and UD, based on MRU [11]. I also described a new comprehensive classification system (Liao’s classification) [15] for LUT and UUT dysfunction (LUUTD) in patients with NB (Table 1).

In Table 1, LUTD is described according to the Guidelines of the European Association of Urology (EAU) [1] and the terminology of the International Continence Society (ICS) [14]. The VUR is graded according to the IRGS. Kidney function is determined by the glomerular filtration rate (GFR), as determined by isotope renography and the serum level of creatinine (Scr). In NB patients, the left and right kidneys usually have different levels of function, therefore unilateral GFR is very important and should be individually noted. The Scr has the same means with the total GFR, and is used to define the decompensation stage of renal function. For NB patients, detrusor fibrosis and thickening secondary to progressive destruction of the bladder wall often result in ureteral strictures within the bladder wall and distal ureteral obstruction, which is an important factor in the etiology of UUTD, including HN and UD, and often results in chronic renal failure. Therefore, this new classification better discriminates among grade changes in UUT function, can provide objective indicators for UUT function, and is an important component of long-term follow-up of conservative and surgical treatment.

**Table 1:** Liao’s comprehensive classification system for lower and upper urinary tract dysfunction (LUUTD) in patients with neurogenic bladder.

Lower Urinary Tract		Upper Urinary Tract
Storage	Voiding	
**1. Bladder function**	**1. Bladder function**	**1. Vesico-ureteral reflux**
(1) Detrusor activity	(1) Detrusor contractility	(1) No
① Normal	① Normal	(2) Yes: Unilateral, bilateral
② Overactive	② Underactive	Degree (left, right)
	③ Acontractile	I
**2. Bladder sensation**		II
(1) Normal	**2. Urethral function**	III
(2) Increased or hypersensitive	(1) Normal	IV
(3) Reduced or hyposensitive	(2) Obstruction	V
(4) Absent	① Urethral overactivity	
	Detrusor external sphincter dyssynergia	**2. Upper urinary tract dilatation: hydronephrosis and ureteral dilatation**
**3. Bladder capacity**	Detrusor bladder neck dyssynergia	(1) No
(1) Normal (300–500 mL)	Sphincter overactivity	(2) Yes: Unilateral, bilateral
(2) High (>500 mL)	Non-relaxing sphincter	Grade (left, right)
(3) Low (<300 mL)	Non-relaxing bladder neck	1
	② Mechanical obstruction	2
**4. Bladder compliance**		3
(1) Normal (20–40 mL/cmH_2_O)		4
(2) High (>40 mL/cmH_2_O)		
(3) Low (<20 mL/cmH_2_O)		**3. Ureteral obstruction in bladder wall**
		(1) No
**5. Urethral function**		(2) Obstruction (left, right)
(1) Normal		
(2) Sphincter acontractility		**4. Renal function**
(3) Incompetent		(1) Normal
		① Unilateral kidney: GRF ≥ 70 mL/min (left, right)
		② Both kidneys: Total GRF ≥ 70 mL/min
① Bladder neck		(2) Renal insufficiency (Total GRF: 50–70 mL/min)
② External sphincter		① Compensatory (Total GRF ≥ 50 mL/min, Scr < 178 μmol/L)
		② Decompensation (Total GRF < 50 mL/min, Scr ≥ 178 μmol/L)

## 3. Management

### 3.1. Intermittent Catheterization

**Figure 7:**
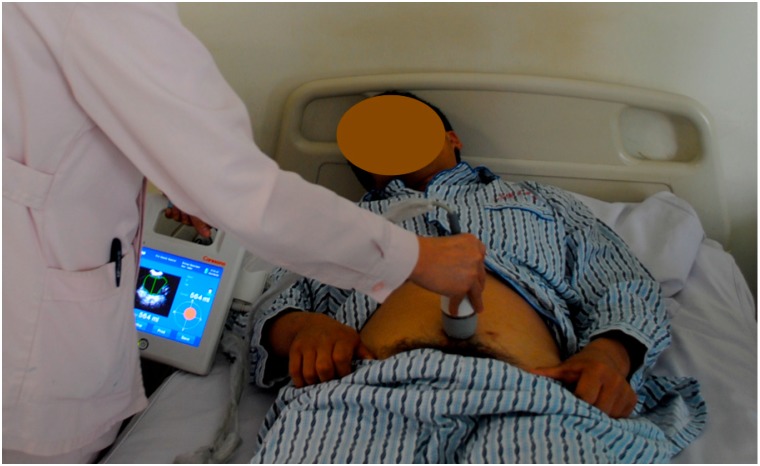
Chinese bladder volume scanner based on US findings to check bladder volume.

Intermittent self- or third-party catheterization is the gold standard for the management of NLUTD [16,17]. The average frequency of catheterizations per day is four to six times and the catheter size should be 12–14 Fr. (French). Less frequent catheterization results in higher catheterization volumes and a higher risk of urinary tract infections. More frequent catheterization increases the risk of cross-infections and other complications. The bladder volume at the time of catheterization should be <400 mL. We developed a Chinese bladder volume scanner based on US findings to check the bladder volume and to guide the patients in performing the clean intermittent catheterization (CIC; Figure 7).

### 3.2. Medication

#### 3.2.1. Anti-Muscarinics

Anti-muscarinic drugs are the first-line choice for treating NLUTD. Anti-muscarinic drugs are the most useful medications available for NLUTD and provide an established approach to managing NDO. Currently, the gold standard treatment for NDO is CIC combined with anti-cholinergic agent therapy. Oxybutynin chloride, trospium chloride, tolterodine tartrate, and propiverine are established, effective, medical treatments. These anti-muscarinic agents are known to be well-tolerated and safe, even during long-term treatment. There is a new oral anti-cholinergic drug, solifenacin, with a high affinity for the M3 muscarinic receptor in the bladder, but data on the impact of solifenacin in patients with NDO are limited. In China, we recently assessed the efficacy and safety of solifenacin in 50 patients with with spinal cord injury (SCI) and NDO, and showed that it increases bladder capacity, decreases bladder leakage volume, and significantly improves the quality of life [18]. Of note, NDO recurs immediately after withdrawal of the anti-muscarinic agents, even after long-term treatment, suggesting that there are no long-lasting therapeutic effects of pharmacologic treatment. Thus, patients with NDO must be permanently treated with anti-muscarinic agents and face lifelong side effects unless completely safe and effective drugs are developed.

#### 3.2.2. Phosphodiesterase Inhibitors (PDE5Is)

PDE5Is have been shown to have significant effects on DO in pilot studies, and may become alternatives or adjuncts to anti-muscarinic treatment in the future, but the data on NDO are limited [19,20]. Recently, nitric oxide (NO) has been shown to participate in the neural pathways that control the LUT. Expression of neural NO synthase (nNOS) can be up-regulated after SCI, and altered nNOS activity may participate in the resulting LUTD. We examined the distribution of nNOS immunoreactivity in rat neurons following SCI, and the impact of nNOS inhibitors, and concluded that the strategic manipulation of NO production could help restore function or reduce undesired functional effects in the LUT [21]. Indeed, we believe that this may become a focus for the development of new pharmacologic interventions, a first step toward which would be the study of nNOS immunoreactivity and the effect of nNOS inhibitor administration in patients at different stages of SCI.

#### 3.2.3. β3-Adrenergic Receptor Agonist

The β3 receptor agonist has recently been introduced and evaluated in patients with overactivity bladder (OAB), but clinical experience in neuro-urologic patients is limited [22,23]. Studies involving the safety and effectiveness in NDO are ongoing. In the future, combined therapy with anti-muscarinics may be an attractive option.

#### 3.2.4. α-Blockers

In some patients with NB, non-selective and selective α-blockers have been partially successful for decreasing bladder outlet resistance, residual urine, and autonomic dysreflexia [24]. Non-selective and selective α-blockers are widely used in China.

### 3.3. Botulinum Toxin A (BTX-A) Injection

BTX-A was introduced to treat NDO by injection into the detrusor muscle on the theoretical basis that BTX-A would temporarily block the pre-synaptic release of acetylcholine from parasympathetic innervation and produce a paralysis of the detrusor smooth muscle. Botulinum toxin causes a long-lasting, but reversible chemical denervation that lasts for approximately nine months. The toxin injections are mapped over the detrusor in a dosage that depends on the preparation used [25,26,27]. Histologic studies have not demonstrated ultrastructural changes after injection [28]. At present, Botox (Allergen) is widely used globally. Lantox is a Chinese BTX-A which is produced by the Lanzhou Institute of Biological Products (Lanzhou, China). We began using this procedure >10 years ago, and the use of Lantox in the management of NDO in patients with spinal cord injury (SCI) achieved similar efficacy, safety, and tolerability profiles to Botox at the same doses [29]. Lantox injection into the detrusor muscle in patients with SCI and NDO significantly improves bladder function, as evidenced by continence and subjective patient satisfaction. The urodynamic parameters measured at the follow-up assessment have shown that the reflex volume, maximum detrusor pressure, bladder compliance, and maximum cystometric capacity are all significantly improved. We have also shown that injection of Lantox into the detrusor muscle significantly reduces urinary tract infections in patients with SCI [30]. We suggest that this reduction in urinary tract infections is related to the decrease in detrusor pressure. The cost of Lantox is one-third that of Botox. A clinical trial on the new indication for Lantox injection into bladder wall will be carried out in China so that this new procedure can be approved for use in clinical practice in China.

Lantox injection into the urethral external sphincter is another effective strategy, which can reduce urethral resistance resulting from a lack of relaxation of the urethral sphincter or DSD, and can decrease the residual volume and improve the voiding efficiency in patients with NLUTD. The efficacy and safety of Lantox intravesical injection for treatment of interstitial cystitis/bladder pain syndrome has also been shown [31].

### 3.4. Neuromodulation

The use of electrical currents for the treatment of LUTD was first reported in 1878 when Saxtorph described intravesical electrostimulation in patients with an acontractile bladder and complete urinary retention. Today, an increasing number of studies are focused on somatic nerve stimulation to treat bladder dysfunction.

#### 3.4.1. Sacral Neuromodulation (SNM)

SNM is an established, minimally invasive, reversible surgical treatment for both LUT and bowel dysfunction [32]. Since the United States Food and Drug Administration (FDA) approved the first SNM implant in the 1990s, >170,000 patients worldwide have used electric stimulation of the sacral nerve to derive benefits from urinary urge incontinence, urgency frequency, non-obstructive urinary retention, fecal incontinence, and other bladder problems. The mechanism of action is not entirely clear, but it is thought that the inhibition of afferent signals interrupts inappropriate detrusor contractions. While it was not thought to be a promising treatment option for patients with NB, our study suggested that patients can benefit from neuromodulation of sacral spinal nerve 3 (S3). Patients with neurologic disease or injury often exhibit multiple symptoms, and while SNM may not resolve all of the symptoms, it may be a good option in combination with other treatments [33,34].

**Figure 8:**
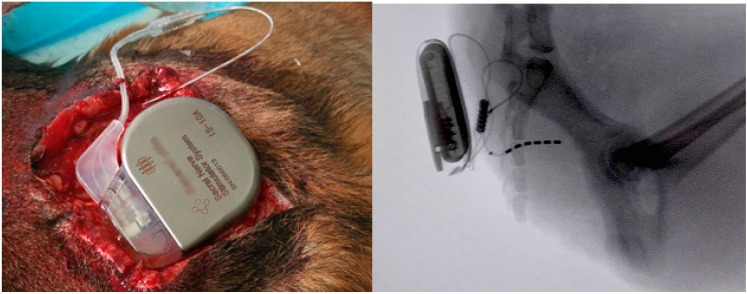
Animal model (dog) for Chinese Sacral Neuromodulation Implant System (SacralStim) in Beijing.

Since the first generation neurostimulator was introduced in 1983, Medtronic (Minneapolis, MN, USA) has dominated the market. Despite clearly documented benefits, technologic advances, and commercial success, the SNM implant is still limited to use in a relatively small proportion of potential candidates in developed countries, and much less so in developing countries. The high cost of the SNM implants has not changed in the last 20 years, despite the increased volume, and is still a major factor limiting market access. To benefit more patients, developing a low-cost, high-performance SNM implant system has long been recognized as an effective and perhaps only means to increase competition and market access, particularly in developing countries. We are currently working on the development of a Chinese SNM implant system called “SacralStim” (Figure 8), and will release the details in the near future.

#### 3.4.2. Pudendal Neuromodulation (PNM)

**Figure 9:**
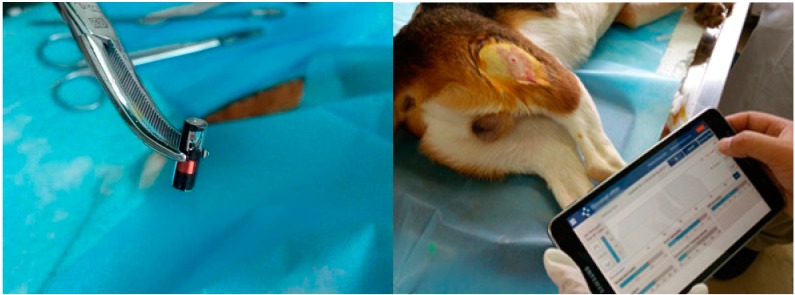
Animal model (dog) for a mini-stimulator (NuStim) without batteries for nerve and muscle stimulation in in Beijing.

Pudendal nerve stimulation (PNS) or PNM is another potential treatment for NDO and has achieved good results in patients with dysfunctional voiding. Our animal studies have shown that stimulation of the pudendal nerve increases bladder capacity only during the early period after SCI [35]. In chronic SCI, the bladder becomes hypertrophic and/or fibrotic, and bladder compliance is significantly altered. We believe that PNM should be used to treat NDO secondary to SCI immediately after other conservative treatments have failed. Further work is needed to determine definitively if early application of PNM also prevents bladder fibrosis, and if so, what the optimal timing is for the treatment. PNM also may be one of the early neuroprotection actions. In an animal model, PNS from the time of SCI inhibits NDO, increases bladder capacity, and delays the progression of bladder fibrosis, leading us to conclude that PNS should be used as early as possible after injury [36]. This finding needs to be translated into clinical practice as soon as possible. There were reports in the literature [37,38] about a battery-powered stimulator (BION, Advanced Bionics Corp., Valencia, CA, USA) approved in Europe for the treatment of urinary urge incontinence by stimulation of the pudendal nerve, but the device was discontinued for unknown reasons. In China, we recently used a similar technique to develop a new mini-stimulator (NuStim) without batteries for nerves and muscles (Figure 9). NuStim is a novel, inductively-powered and -controlled micro-stimulator for chronic electrical stimulation. NuStim is small enough (3 mm in diameter × 10 mm in length) to be implanted percutaneously into a muscle and close to the nerve (pudendal nerve) via a simple insertion tool consisting of a dilator and a sheath. NuStim was provided with power by a radio frequency (RF)-cushion. NuStim therapy will be used to perform neuromodulation for the nerve and muscles, such as PNM for OAB and pelvic floor muscles (PFM) stimulation for stress urinary incontinence (SUI). Currently a number of different SUI procedures exist, including Kegel exercises of PFM and the midurethral sling procedures, for example, tension-free vaginal tapes (TVT). The long-term failure rate and complications of these sling procedures have been brought to our attention [39]; there may be few suitable alternatives clinically. In the conservative management, Kegel exercises are effective to treat SIU by strengthening the PFM, but some patients lack the ability to voluntarily contract these muscles fully and/or are unwilling to do the exercises frequently enough to obtain the full benefit. Under these backgrounds, we have developed this injectable and wireless NuStim that could be implanted percutaneously into the PFM. Electrical stimulation of the intramuscular motor axons generates strong contractions of the PFM and results in a similar outcome to Kegel exercises. Compared to conventional surgical treatments for SUI, we hypothesize this innovation will be less invasive and have fewer post-operative and long-term complications, while achieving significant reduction of urinary leakage in patients with mild or moderate SUI. NuStim is undergoing animal trials.

#### 3.4.3. Percutaneous Tibial Nerve Stimulation (PTNS)

**Figure 10:**
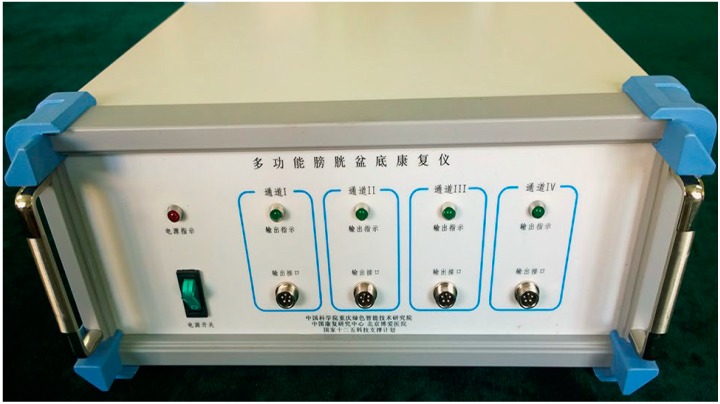
Bladder-Pelvic Stimulator.

SNM and PNM, as practiced today, are invasive and require surgery to implant an electrical stimulator and an electrode. We have recently found, however, that percutaneous tibial nerve stimulation (PTNS) using adhesive skin-surface electrodes placed on the ankle is effective in treating NDO in 50 patients with spinal cord injuries, and we developed a Bladder-Pelvic Stimulator (Figure 10) [18].

A study in cats revealed that inhibition of OAB by PTNS depends on the activation of opioid receptors. This suggests a novel treatment strategy for OAB (combining PTNS with a low dose of tramadol, a narcotic-like pain reliever). PTNS is minimally invasive, of potentially high efficacy, and is likely to have few adverse effects [40].

#### 3.4.4. Foot Stimulation

A previous study showed that foot stimulation can delay the bladder filling sensation and increase bladder volume in healthy humans without OAB. We completed a study to determine whether or not electrical stimulation of somatic afferent nerves in the foot can increase bladder capacity in patients with NB after sigmoid cystoplasty. We showed that the bladder volume was increased from 279.4 to 361.1 mL after stimulation using the Bladder-Pelvic Stimulator [41]. A branch of the tibial nerve courses through the foot, which likely explains this effect. Given the non-invasiveness of this stimulation method, a clinical trial could easily be conducted.

### 3.5. Urinary Tract Reconstruction Surgery

#### 3.5.1. Augmentation Enterocystoplasty

Urinary infection and VUR, detrimental effects on the UUT secondary to high bladder pressure, are common and devastating problems for patients with NB. Preservation of the UUT is the most important goal in any type of LUT reconstruction. The gold standard treatment for urinary infection and VUR is augmentation enterocystoplasty, which aims to create a reservoir with a large capacity and good compliance, while preserving the UUT, thus allowing for socially-acceptable continence. All segments of the bowel have been utilized for augmentation enterocystoplasty. The sigmoid has advantages, including its anatomic proximity to the bladder, as well as a thick muscular wall, large lumen, and abundant mesentery, which together guarantees adequate capacity and maneuverability for the bladder. We retrospectively reviewed a total of 78 augmentation enterocystoplasties performed between 2005 and 2011, and identified 47 patients who underwent sigmoidocolocystoplasty alone or in combination with ureteral re-implantation. Sigmoidocolocystoplasty was shown to be a safe and effective treatment for neurogenic bladder, and the concomitant ureteral re-implantation was beneficial for patients with a long history of illness. Moreover, in the majority of patients the procedure resolved urinary infection under CIC [42]. Consequently, sigmoidocolocystoplasty is included in our routine neuro-urologic practice, and we have performed sigmoidocolocystoplasty in >250 patients to date.

To improve our ability to make clinical decisions and identify changes in UUTD pre- and post-treatment, we recently introduced two innovations (an UUTD grading system based on MRU [11] and a comprehensive classification system [15] for LUUTD in patients with NB). Evaluating the midterm outcomes using these tools demonstrated conclusively that augmentation enterocystoplasty provides effective and safe UUT protection in patients with moderate and severe upper urinary tract deterioration and renal function impairment resulting from VUR and UUTD [43].

#### 3.5.2. Tissue-Engineering Bladder Augmentation

The presence of gastrointestinal segments in the urinary tract has been associated with many complications, such as adhesive intestinal obstruction, metabolic disturbances, urolithiasis, excessive mucous production, and malignant diseases. Composite cystoplasty and the use of substitution materials have been proposed as a means of overcoming these complications, and we have been testing tissue-engineering technology during cystoplasty in animal experiments [44]. Specifically, we have used small intestine submucosa (SIS) as a bladder wall replacement in a rabbit augmentation model. Histologically, the SIS-regenerated bladders resemble normal bladder, and all three tissue layers (mucosa with submucosa, smooth muscle, and serosa) are present. In an *in vitro* study of the detrusor strip there were no significant differences in autorhythmicity and contractility between regenerated and normal detrusor muscles. An immunohistochemical analysis has indicated that the quantity of α-actin developed to a normal level and urodynamic testing showed that compliance remained stable post-operatively, and the volume increased significantly.

On the basis of these animal experiments, we explored the use of a SIS scaffold for bladder augmentation in neurogenic bladder patients [45]. Preliminary data from 14 cases have shown that cystoplasty with SIS can improve the functionality of bladders in NB patients. No metabolic consequences were noted, no urinary calculi were observed, and renal function was preserved. The similar and promising results have been noted previously with polyglycolic acid and other materials, as well as with seeded and non-seeded scaffolds, only to demonstrate later that these patches contracted, capacity diminished, and pressures rose, leading to the necessity of an enteric augmentation [46,47,48]. We will continue our careful follow-up of this cohort for several more years to determine if these moderate-term good results are maintained. Whether or not non-seeded scaffolds are a better long-term source than cell-seeded constructs remains to be seen. We do not currently recommend bladder augmentation using SIS as a substitute for enterocystoplasty; nevertheless, this tissue-engineering technology does provide a potentially viable option for urinary tract reconstruction in NB patients.

### 3.6. Artificial Urinary Sphincter (AUS)

Artificial urinary sphincter (AUS) implantation plays an important role in urinary tract reconstruction (UTR) for patients with complex urinary incontinence (UI), including neurogenic UI [49,50,51]. Patients with NB have low bladder outlet resistance and AUS can offer such patients the possibility of spontaneous voiding [51]. We retrospectively reviewed our institution data and selected 30 UI patients who had undergone AUS placement during UTR. Nine patients (30%) had neurogenic UI, 17 (56.7%) had post-traumatic sphincter incontinence, and four (10.33%) had post-prostatectomy incontinence (PPI). There was a significant reduction in pad count leading to a dry rate of 46.7% after surgery. The primary sources for AUS implantation in China are unique, and the procedure is an effective treatment as a key procedure for UTR, especially in neurogenic UI cases. Cuff erosion, urethral atrophy, and mechanical device failure are the well-known complications during the long-term follow up, all of which require revision surgery.

### 3.7. New Treatments

#### 3.7.1. Stem Cell Transplantation

The mature central nervous system (CNS) cannot generate new neurons and glial cells. Consequently, recovery of bladder function is limited following SCI; however, a study suggested that transplanted neural progenitor cells or other stem cells can promote recovery of bladder function through regeneration of the injury site [52]. In most of these studies, stem cells have been injected directly into the lesion, which carries the risk of further injury to the spinal cord. We have taken the alternative approach of intravenous injection of bone marrow stromal cells (BMSCs). These cells survive in lumbar vertebrae 3 and 4 (L_3_–L_4_) for at least four weeks, and we observed some improvements in LUT function in rats with SCI. Although the study was preliminary, it does suggest that intravenous transplantation of BMSCs has potential for the treatment of LUT dysfunction after SCI in humans [53]. BMSCs also may become one of the early neuroprotective strategies.

#### 3.7.2. Gene Therapy

Gene therapy has also been explored for the treatment of organ dysfunction secondary to SCI, including NB disorder. It is known that suppressing *N*-methyl-d-aspartate receptors (NMDARs) can improve DO in rats with SCI. We took a gene therapy approach to express kynurenic acid, the endogenous antagonist of NMDARs. We transferred the gene for human kynurenine aminotransferase II (KAT II), which synthesizes kynurenic acid, to a replication-defective herpes simplex virus vector (HSVrd) and injected the HSVrd into the bladder walls of rats. The vector was transported to the L6-S1 dorsal root ganglia, the expression of KAT II was up-regulated, and there was an improvement in DO and voiding efficiency. We also confirmed by whole-cell patch clamping of cultured rat neurons of the L6-S1 dorsal root ganglia that NMDARs are blocked by kynurenic acid present in the extracellular solution or delivered by vector-mediated gene transfer of KAT II. Therefore, HSVrd-mediated KAT II inhibits DO in rats with SCI, possibly through suppression of NMDARs in bladder afferent pathways [54]. Currently we are continuing to inject HSVrd-mediated KAT II into the urethral sphincter of rats in order to reduce urethral resistance and treat DSD in rats with SCI. These results are awaiting translation into clinical practice.

### 3.8. Guidelines on Neurogenic Bladder (NB) in China

The Chinese Urologic Association of (CUA) published the first NB guidelines, chaired by Liao in 2011, with updates in 2014 and 2015 [55,56]. The Chinese NB guidelines provide information for clinical practitioners on the incidence, definition, diagnosis, therapy, and follow-up of NB. The guidelines represent the current opinion of experts from China in this field, and thus represent a state-of-the-art reference for all clinicians in China.

## 4. Conclusions

In summary, the evaluation and management of NB is a challenge to contemporary medical science and neuroscience. The evaluation and management of NB includes traditional principles and new technologic progress. In the field of neuro-urology, translational medicine has a long way to go, but we are determined to meet the many challenges. We are looking forward in this field in China. There are several promising new treatments for NB, although some treatments are still at the animal experimental stage. Further work will focus on combining two or more methods to enhance treatment, and on translating these techniques into clinical practice in humans. With respect to pharmacology, the focus will be on relaxation of the afferent side of the detrusor muscle and new targets of the CNS. Ongoing global trials using BTX-A will help establish standardized injection protocols. NB is a good indication for neuromodulation based on our experience. Other potentially fruitful avenues include tissue engineering, the use of stem cells, gene therapy, and other neuroprotective strategies. The overall focus of these approaches is on restorative therapy, while avoiding destructive surgery, aimed at promoting nerve recovery and protecting UUT function.

## References

[1] Stohrer M., Blok B., Castro-Diaz D., Chartier-Kastler E., del Popolo G., Kramer G., Pannek J., Radziszewski P., Wyndaele J.J. (2009). EAU guidelines on neurogenic lower urinary tract dysfunction. Eur. Urol..

[2] Blok B.F., Willemsen A.T.M., Holstege G. (1997). A PET study on brain control of micturition in humans. Brain.

[3] Athwal B.S., Berkley K.J., Hussain I., Brennan A., Craggs M., Sakakibara R., Frackowiak R.S., Fowler C.J. (2001). Brain responses to changes in bladder volume and urge to void in healthy men. Brain.

[4] Zhang H., Reitz A., Kollias S., Summers P., Curt A., Schurch B. (2005). An fMRI study of the role of suprapontine brain structures in the voluntary voiding control induced by pelvic floor contraction. Neuroimage.

[5] Gao Y., Liao L.M., Blok B.F. (2015). A resting-state functional MRI study on central control of storage: Brain response provoked by strong desire to void. Int. Urol. Nephrol..

[6] Schaefer W., Abrams P., Liao L.M., Mattiasson A., Pesce F., Spangberg A., Sterling A.M., Zinner N.R., van Kerrebroeck P. (2002). Good urodynamic practices: Uroflowmetry, filling cystometry, and pressure-flow studies. Neurourol. Urodyn..

[7] Liao L.M., Schaefer W. (2014). Quantitative quality control during urodynamic studies with TVRs for cystometry in men with lower urinary tract symptoms suggestive of benign prostatic hyperplasia. Int. Urol. Nephrol..

[8] Liao L.M., Schaefer W. (2014). Qualitative quality control during urodynamic studies with TSPs for cystometry in men with lower urinary tract symptoms suggestive of benign prostatic hyperplasia. Int. Urol. Nephrol..

[9] Liao L.M., Schaefer W. (2007). Effects of retrospective quality control on pressure-flow data with computer-based urodynamic systems from men with benign prostatic hyperplasia. Asian J. Androl..

[10] Stoehrer M., Goepel M., Kondo A., Kramer G., Madersbacher H., Millard R., Rossier A., Wyndaele J.J. (1999). The standardization of terminology in neurogenic lower urinary tract dysfunction: With suggestions for diagnostic procedures. International continence Society Standardization Committee. Neurourol. Urodyn..

[11] Liao L.M., Zhang F., Chen G. (2014). New grading system for upper urinary tract dilation using magnetic resonance urography in patients with neurogenic bladder. BMC Urol..

[12] Duckett J.W., Bellinger M.F. (1982). A plea for standardized grading of vesicoureteral reflux. Eur. Urol..

[13] Madersbacher H. (1990). The various types of neurogenic bladder dysfunction: An update of current therapeutic concepts. Paraplegia.

[14] Abrams P., Cardozo L., Fall M., Griffiths D., Rosier P., Ulmsten U., van Kerrebroeck P., Victor A., Wein A. (2002). The standardization of terminology of lower urinary tract function. Neurourol. Urodyn..

[15] Liao L.M. (2015). A new comprehensive classification system for both lower and upper urinary tract dysfunction in patients with neurogenic bladder. Urol. Int..

[16] Guttmann L., Frankel H. (1966). The value of intermittent catheterisation in the early management of traumatic paraplegia and tetraplegia. Paraplegia.

[17] Wyndaele J.J. (2002). Intermittent catheterization: Which is the optimal technique?. Spinal Cord.

[18] Chen G., Liao L.M., Li Y. (2015). The possible role of percutaneous tibial nerve stimulation using adhesive skin surface electrodes in patients with neurogenic detrusor overactivity secondary to spinal cord injury. Int. Urol. Nephrol..

[19] Angulo J., Cuevas P., Fernández A., la Fuente J.M., Allona A., Moncada I., Sáenz de Tejada I. (2012). Tadalafil enhances the inhibitory effects of tamsulosin on neurogenic contractions of human prostate and bladder neck. J. Sex Med..

[20] Behr-Roussel D., Oger S., Caisey S., Sandner P., Bernabé J., Alexandre L., Giuliano F. (2011). Vardenafil decreases bladder afferent nerve activity in unanesthetized, decerebrate, spinal cord-injured rats. Eur. Urol..

[21] Zhang F., Liao L.M., Ju Y., Song A., Liu Y. (2011). Neurochemical plasticity of nitric oxide synthase isoforms in neurogenic detrusor overactivity after spinal cord injury. Neurochem. Res..

[22] Herschorn S., Barkin J., Castro-Diaz D., Frankel J.M., Espuna-Pons M., Gousse A.E., Stölzel M., Martin N., Gunther A., van Kerrebroeck P. (2013). A phase III, randomized, double-blind, parallel-group, placebo-controlled, multicentre study to assess the efficacy and safety of the β_3_ adrenoceptor agonist, mirabegron, in patients with symptoms of overactive bladder. Urology.

[23] Nitti V.W., Rosenberg S., Mitcheson D.H., He W., Fakhoury A., Martin N.E. (2013). Urodynamics and safety of the β_3_-adrenoceptor agonist mirabegron in males with lower urinary tract symptoms and bladder outlet obstruction. J. Urol..

[24] Abrams P., Amarenco G., Bakke A., Buczyński A., Castro-Diaz D., Harrison S., Kramer G., Marsik R., Prajsner A., Stöhrer M. (2003). Tamsulosin: Efficacy and safety in patients with neurogenic lower urinary tract dysfunction due to suprasacral spinal cord injury. J. Urol..

[25] Schurch B., de Sèze M., Denys P., Chartier-Kastler E., Haab F., Everaert K., Plante P., Perrouin-Verbe B., Kumar C., Fraczek S. (2005). Botulinum toxin type a is a safe and effective treatment for neurogenic urinary incontinence: Results of a single treatment, randomized, placebo controlled 6-month study. J. Urol..

[26] Cruz F., Herschorn S., Aliotta P., Brin M., Thompson C., Lam W., Daniell G., Heesakkers J., Haag-Molkenteller C. (2011). Efficacy and safety of onabotulinumtoxinA in patients with urinary incontinence due to neurogenic detrusor overactivity: A randomised, double-blind, placebo-controlled trial. Eur. Urol..

[27] Grosse J., Kramer G., Stöhrer M. (2005). Success of repeat detrusor injections of botulinum a toxin in patients with severe neurogenic detrusor overactivity and incontinence. Eur. Urol..

[28] Haferkamp A., Schurch B., Reitz A., Krengel U., Grosse J., Kramer G., Schumacher S., Bastian P.J., Büttner R., Müller S.C. (2004). Lack of ultrastructural detrusor changes following endoscopic injection of botulinum toxin type a in overactive neurogenic bladder. Eur. Urol..

[29] Chen G., Liao L.M. (2011). Injections of Botulinum Toxin A into the detrusor to treat neurogenic detrusor overactivity secondary to spinal cord injury. Int. Urol. Nephrol..

[30] Jia C., Liao L.M., Chen G., Sui Y. (2013). Detrusor botulinum toxin A injection significantly decreased urinary tract infection in patients with traumatic spinal cord injury. Spinal Cord.

[31] Gao Y., Liao L.M. (2015). Intravesical injection of botulinum toxin A for treatment of interstitial cystitis/bladder pain syndrome: 10 years of experience at a single center in China. Int. Urogynecol. J..

[32] Wollner J., Hampel C., Kessler T.M. (2012). Surgery Illustrated—Surgical atlas sacral neuromodulation. BJU Int..

[33] Kessler T.M., La Framboise D., Trelle S., Fowler C.J., Kiss G., Pannek J., Schurch B., Sievert K.D., Engeler D.S. (2010). Sacral neuromodulation for neurogenic lower urinary tract dysfunction: Systematic review and meta-analysis. Eur. Urol..

[34] Chen G., Liao L.M. (2014). Sacral neuromodulation for neurogenic bladder and bowel dysfunction with multiple symptoms secondary to spinal cord disease. Spinal Cord.

[35] Chen G., Liao L., Dong Q., Ju Y. (2012). The inhibitory effects of pudendal nerve stimulation on bladder overactivity in spinal cord injury dogs: Is early stimulation necessary?. Neuromodulation.

[36] Li P., Liao L., Chen G., Zhang F., Tian Y. (2013). Early low-frequency stimulation of the pudendal nerve can inhibit detrusor overactivity and delay progress of bladder fibrosis in dogs with spinal cord injuries. Spinal Cord.

[37] Bosch J.L. (2005). The bion device: A minimally invasive implantable ministimulator for pudendal nerve neuromodulation in patients with detrusor overactivity incontinence. Urol. Clin. N. Am..

[38] Groen J., Amie C.L., Bosch J.L.H.R. (2005). Chronic pudendal nerve neuromodulation in women with idiopathic refractory detrusor overactivity incontinence: Results of a pilot study with a novel minimally invasive implantable mini-stimulator. Neurourol. Urodyn..

[39] Novara G., Artibani W., Barber M.D., Chapple C.R., Costantini E., Ficarra V., Hilton P., Nilsson C.G., Waltregny D. (2010). Updated systematic review and meta-analysis of the comparative data on colposuspensions, pubovaginal slings, and midurethral tapes in the surgical treatment of female stress urinary incontinence. Eur. Urol..

[40] Zhang F., Mally A.D., Ogagan P.D., Shen B., Wang J., Roppolo J.R., de Groat W.C., Tai C. (2012). Inhibition of bladder overactivity by a combination of tibial neuromodulation and tramadol treatment in cats. Am. J. Physiol. Ren. Physiol..

[41] Chen G., Liao L.M., Miao D. (2015). Electrical stimulation of somatic afferent nerves in the foot increases bladder capacity in neurogenic bladder patients after sigmoid cystoplasty. BMC Urol..

[42] Zhang F., Liao L.M. (2012). Sigmoidocolocystoplasty with ureteral reimplantation for treatment of neurogenic bladder. Urology.

[43] Liao L.M., Zhang F., Chen G. (2014). Midterm outcomes of protection for upper urinary tract function by augmentation enterocystoplasty in patients with neurogenic bladder. Int. Urol. Nephrol..

[44] Wang Y., Liao L.M. (2014). Histologic and functional outcomes of small intestine submucosa-regenerated bladder tissue. BMC Urol..

[45] Zhang F., Liao L.M. (2014). Tissue engineered cystoplasty augmentation for treatment of neurogenic bladder using small intestinal submucosa: An exploratory study. J. Urol..

[46] Kajbafzadeh A.M., Tourchi A., Mousavian A.A., Rouhi L., Tavangar S.M., Sabetkish N. (2014). Bladder muscular wall regeneration with autologous adipose mesenchymal stem cells on three-dimensional collagen-based tissue-engineered prepuce and biocompatible nanofibrillar scaffold. J. Pediatr. Urol..

[47] Lai J.Y., Chang P.Y., Lin J.N. (2005). Bladder autoaugmentation using various biodegradable scaffolds seeded with autologous smooth muscle cells in a rabbit model. J. Pediatr. Surg..

[48] Kwon T.G., Yoo J.J., Atala A. (2008). Local and systemic effects of a tissue engineered neobladder in a canine cystoplasty model. J. Urol..

[49] Léon P., Chartier-Kastler E., Rouprêt M., Ambrogi V., Mozer P., Phé V. (2015). Long-term functional outcomes after artificial urinary sphincter (AMS 800^®^) implantation in men with stress urinary incontinence. BJU Int..

[50] Kim S.P., Sarmast Z., Daignault S., Faerber G.J., McGuire E.J., Latini J.M. (2008). Long-term durability and functional outcomes among patients with artificial urinary sphincters: A 10-year retrospective review from the University of Michigan. J. Urol..

[51] Lai H.H., Hsu E.I., The B.S., Butler E.B., Boone T.B. (2007). 13 years of experience with artificial urinary sphincter implantation at Baylor College of Medicine. J. Urol..

[52] Neuhuber B., Barshinger A.L., Paul C., Shumsky J.S., Mitsui T., Fischer I. (2008). Stem cell delivery by lumbar puncture as a therapeutic alternative to direct injection into injured spinal cord. J. Neurosurg. Spine.

[53] Hu Y., Liao L.M., Ju Y.H., Fu G., Zhang H.Y., Wu H.X. (2012). Intravenously transplanted bone marrow stromal cells promote recovery of lower urinary tract function in rats with complete spinal cord injury. Spinal Cord.

[54] Jia C., Yoshimura N., Liao L. (2014). Herpes simplex virus vector-mediated gene transfer of kynurenine aminotransferase improves detrusor overactivity in spinal cord-injured rats. Gene Ther..

[55] Liao L.M., Song B., Na Y.Q., Ye Z.Q., Sun G. (2011). Guidelines on neurogenic bladder. Chinese Guidelines on Urologic Diseases.

[56] Liao L.M., Na Y.Q., Ye Z.Q., Sun Y.H., Sun G. (2014). Guidelines on neurogenic bladder. Chinese Guidelines on Urologic Diseases.

